# Investigation of reactions between trace gases and functional CuO nanospheres and octahedrons using NEXAFS-TXM imaging

**DOI:** 10.1038/srep17729

**Published:** 2015-12-03

**Authors:** Katja Henzler, Axel Heilemann, Janosch Kneer, Peter Guttmann, He Jia, Eckhard Bartsch, Yan Lu, Stefan Palzer

**Affiliations:** 1 Institute for Soft Matter and Functional Materials, Helmholtz-Zentrum Berlin für Materialien und Energie GmbH, Hahn-Meitner-Platz 1, 14109 Berlin, Germany; 2 Institut für Makromolekulare Chemie, University of Freiburg, 79104 Freiburg, Germany; 3 Institut für Physikalische Chemie, University of Freiburg, 79104 Freiburg, Germany; 4 Laboratory for Gas Sensors, Department of Microsystems Engineering, University of Freiburg, Georges-Köhler Allee 102, 79110 Freiburg

## Abstract

In order to take full advantage of novel functional materials in the next generation of sensorial devices scalable processes for their fabrication and utilization are of great importance. Also understanding the processes lending the properties to those materials is essential. Among the most sought-after sensor applications are low-cost, highly sensitive and selective metal oxide based gas sensors. Yet, the surface reactions responsible for provoking a change in the electrical behavior of gas sensitive layers are insufficiently comprehended. Here, we have used near-edge x-ray absorption fine structure spectroscopy in combination with x-ray microscopy (NEXAFS-TXM) for *ex-situ* measurements, in order to reveal the hydrogen sulfide induced processes at the surface of copper oxide nanoparticles, which are ultimately responsible for triggering a percolation phase transition. For the first time these measurements allow the imaging of trace gas induced reactions and the effect they have on the chemical composition of the metal oxide surface and bulk. This makes the new technique suitable for elucidating adsorption processes *in-situ* and under real operating conditions.

In recent years micro- and nano-scaled particles of various morphologies and sizes have been investigated in order to utilize their unique properties for a multitude of technological applications, including energy conversion and storage^1^, plasmonics^2^, drug delivery^3^, catalysis^4^,^5^ and gas sensing^6^. Among the impediments preventing a quick commercial adaptation of groundbreaking research results is the lack of scalable interfaces between the nano-regime and macroscopic equipment. One appealing technology in that regard is the inkjet printing process, which provides a precise and scalable method to deposit functional nanoparticles onto arbitrary structures. Using this technique colloidal suspensions of metal oxide inks may be deposited and used as a gas sensitive layer on low-power consuming, micro-machined silicon-based structures. This enables the investigation of the gas sensitive behavior of metal oxide particles as a function of size, shape and temperature. In light of the need of highly sensitive and selective gas sensor solutions for advanced analytical applications the understanding of the underlying processes as well as the possibility to scale the production processes are of primary concern.

Until now, most works are focusing on using n-type metal oxide semiconductors, such as SnO_2_, WO_3_ or ZnO for gas sensors. Only limited work has been reported on p-type semiconducting gas sensors based on materials such as Cu_2_O and CuO. However, p-type gas sensitive materials show significant surface reactivity with reducing and oxidizing gases albeit at lower operating temperatures as compared to n-type materials and consequently they might play a pivotal role in future, low-energy consuming gas sensing devices. In the past Cu_2_O and CuO nanostructures have been fabricated using numerous approaches, few of which are compatible with large-scale production. And while gas-surface interactions are proposed to explain the gas sensitive behavior of metal oxide layers, analytical tools for directly observing adsorption processes are scarce^7^. In this regard, techniques of x-ray absorption spectroscopy offer unique possibilities, but have so far not been used to directly monitor the chemical structure of the gas sensitive material itself. In previous works the oxidation state of platinum upon gas exposure has been investigated^8^,^9^ in order to study its effect on the gas sensitive material. To monitor the gas sensitive material directly Cu_2_O as well as CuO offer ideal conditions to demonstrate a novel approach combining near-edge x-ray absorption fine structure spectroscopy with x-ray microscopy NEXAFS-TXM^10^ and gas sensitive characterizations. This technique is able to probe both, the O-*K*-edge as well as the Cu-*L*_*2,3*_-edge^11^ to reveal specific surface processes upon exposure to a trace gas.

Here, we focus on the reactions between the highly toxic trace gas hydrogen sulfide (H_2_S) and CuO to highlight the capabilities of the technique. We employ the novel approach to track the fundamental changes in CuO caused by the interaction with H_2_S using the NEXAFS-TXM method to shed light onto fundamental surface processes. To address both applied as well as fundamental issues of next generation metal oxide based sensors we present a new route for synthesizing Cu_2_O nanoparticles with high yield, and demonstrate how to interface and to use them as sensor material. Using on-chip annealing the Cu_2_O nanoparticles may be converted to CuO. Interestingly, the shape of the nanoparticles remains the same, even though the crystal structure changes from cubic to monoclinic and the oxygen content increases to about 50%. Hence, the here presented route allows for producing either CuO or Cu_2_O nanoparticles, controlling their shape and size and using their specific gas sensitive characteristics. The Cu_2_O nanoparticles are dispersed in a solution that is subsequently used as ink for depositing the nanoparticles employing an inkjet printing system. The solvent of the ink is then evaporated without leaving contaminations in the sensing layer, i.e. without affecting the gas sensing properties after deposition. The presented approach is able to pave the way towards a new generation of gas sensitive materials with tailor-made properties and reproducible, stable base-line resistances. The gas sensing properties of the CuO particles are investigated and in addition the *ex-situ* NEXAFS-TXM measurements reveal the changes of the chemical composition due to the annealing process as well as those caused by exposure of Cu_2_O and CuO to H_2_S. The induced chemical changes of the Cu_2_O/CuO particles by H_2_S are irreversible at room temperature. Therefore, it was possible to map all stages of the surface reactions between H_2_S and Cu_2_O/CuO *ex-situ* in the high vacuum setup. This demonstrates the suitability of the NEXAFS-TXM method to provide a spatially resolved analysis of the chemical bonds formed during gas-surface interaction, which are at the heart of gas detection using semiconducting metal-oxides as functional material.

## Results and Discussion

### Large-scale synthesis route for well-defined Cu_2_O nanoparticles

The synthesis of Cu_2_O nanoparticles relies on a precipitation reaction in alkaline media:


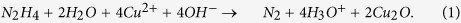


**Figure 1:**
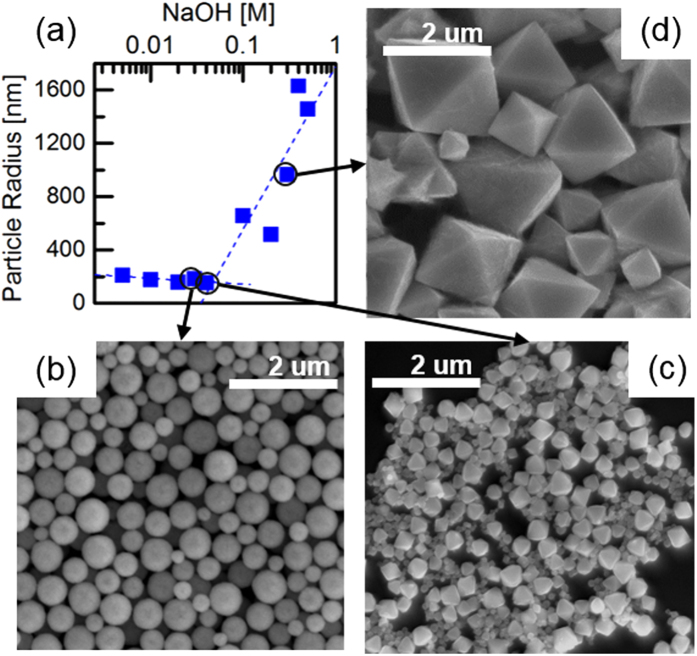
Both the shape as well as the particle size of the Cu_2_O particles crucially depend on the NaOH concentration. **(a)** The particle radius has been determined using dynamic light scattering. **(b)** Concentrations up to 0.03 M NaOH result in spherical particles with a diameter around 200 nm and a reasonably narrow particle size distribution. **(c)** Above 0.04 M NaOH the shape changes to octahedrons and **(d)** further increasing the NaOH concentration results in larger particle sizes. **(b–d)** show scanning electron microscopy (SEM) images for particles produced using 0.03, 0.04 and 0.3 M NaOH, respectively.

In order to increase the yield and to control the shape and the size of the particles, NaOH has been added to the reaction^12^,^13^. Besides the positive effect on the yield, NaOH may be used to control the morphology and the size of the resulting Cu_2_O particles. To this end, the NaOH concentration has been varied from 0.002–0.5 M NaOH. Up to a NaOH concentration of 0.04 M, the particle size shows a slight decrease of the hydrodynamic radius from about 240 nm to about 160 nm observed by dynamic light scattering^14^,^15^,^16^ shown in Fig. 1a. For these samples a spherical shape and a reasonably narrow size distribution can be inferred from scanning electron microscopy (SEM) pictures as exemplarily depicted in Fig. 1b. A sharp transition occurs for NaOH concentrations exceeding 0.04 M (Fig. 1a), when the shape of Cu_2_O particles changes from spherical to octahedral. The addition of NaOH into the reaction solution has been proved to provide the necessary alkalinity for the use of N_2_H_4_ as reducing agent^17^,^18^. Two factors are mainly influencing the morphology evolution of Cu_2_O nanoparticles: One is the capping agent, Polyvinylpyrrolidone (PVP). As a surfactant, PVP is preferentially adsorbed onto the {111} planes of the Cu_2_O crystals, which can decrease the surface energy of the {111} surface and reduce its growth rate. This favors the Cu_2_O crystal growth into octahedral structures^19^,^20^. Another factor is the amount of NaOH in the system. During the reaction, Cu(OH)_2_ is produced by NaOH reacting with Cu^2+^ ions, which is the key intermediate. The production of Cu(OH)_2_ can decrease the reduction rate of Cu_2_O, which slows down the growth rate of Cu_2_O crystals indirectly. Without addition of NaOH, the crystal growth rate is too fast to be controlled by PVP. Adding NaOH to the reaction, the crystal growth rate will be slowed down. In this case, the previously mentioned formation of Cu_2_O octahedrons due to the presence of PVP occurs. Moreover, continuously increasing the concentration of NaOH, [Cu(OH)_4_]^2−^ can be formed via the complexation of Cu^2+^ with OH^−^, which will increase the edge lengths of the octahedrons as shown in Fig. 1c,d, a similar result also has been found by W. Zhu and his co-workers^13^.

### Characterization of the as-synthesized particles and solid-state transformation to CuO nanoparticles

**Figure 2:**
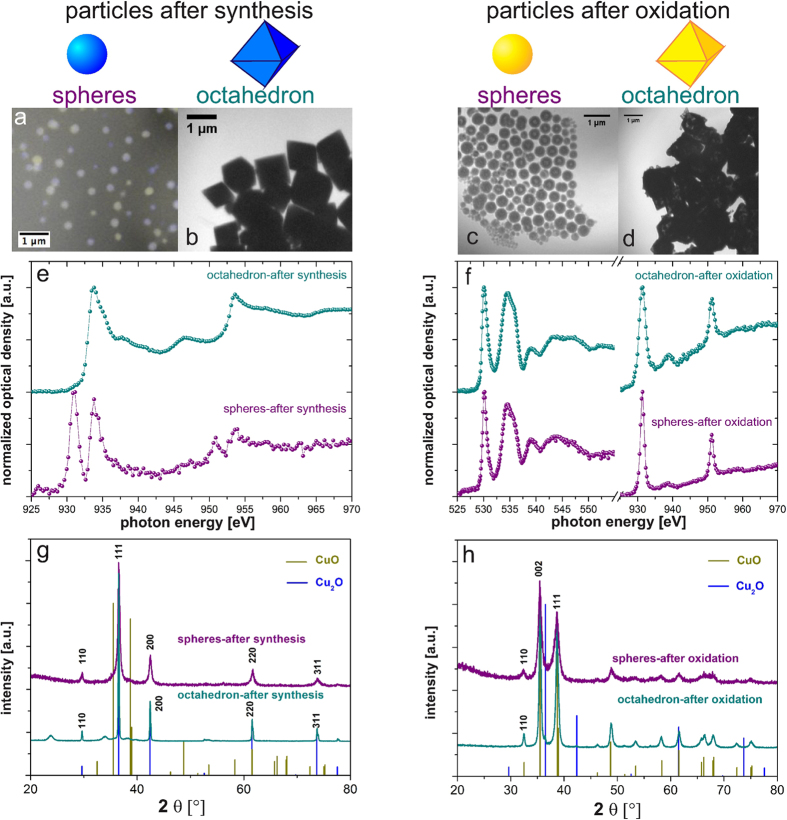
**(a)** TXM-micrograph of the Cu_2_O spheres after synthesis: overlay of 930.8 eV (yellow) and 933.8 eV (blue) represents the chemical inhomogeneity of the sample. **(b)** TXM-micrograph of the Cu_2_O octahedron after synthesis (933.8 eV). **(c)** TXM-micrograph of the CuO spheres after oxidation (532.7 eV). **(d)** TXM-micrograph of the CuO octahedron after oxidation (532.7 eV). **(e)** NEXAFS spectra at the Cu-*L*_*2,3*_-edge of the Cu_2_O nanoparticles after synthesis. **(f)** NEXAFS spectra at the O-*K*-edge and Cu-*L*_*2,3*_-edge of the CuO nanoparticles after oxidation. In the XRD pattern, the measured intensity is plotted versus the scattering angle 2Θ. **(g)** XRD pattern of Cu_2_O nanoparticles after synthesis. **(h)** XRD pattern of CuO nanoparticles after oxidation. The reference values for the expected Bragg peaks for Cu_2_O and CuO are depicted via the δ–function at the corresponding angles.

Using the scalable wet chemistry process it is possible to control shape and size of the Cu_2_O particles. The deposition of the particles is achieved by employing a DIMATIX DMP-2831^21^ system. Tuning the rheological properties by adding polyethylene glycol (PEG) 400 is necessary to match the requirements imposed by the inkjet printing system. Due to a solid state phase transformation from Cu_2_O to CuO at temperatures above 250 °C and ambient pressure it is furthermore possible to convert the so-produced particles using an on-chip annealing technique. Here we used elevated temperatures for one hour, which results in pure CuO particles of the same shape as the Cu_2_O particles. In order to examine the long-range as well as the short-range structure of the nanoparticles NEXAFS spectra and x-ray diffraction (XRD) pattern have been recorded for both spherical and octahedral samples before and after annealing. The results are presented in Fig. 2.

Figure 2a–d shows the TXM-micrographs of the respective samples. The O-*K*-edge of the particles directly after synthesis cannot be investigated due to the remaining PEG, because they have been inkjet printed in order to ensure complete correspondence between NEXAFS analysis and gas sensitive characterization. However, the Cu-*L*_*2,3*_ edge shown in Fig. 2e reveals the differences in the near range order when comparing spherical and octahedron particles after synthesis. The Cu-*L*_2,3_-edge reveals that the spherical particles consist of a mixture of Cu^+^ and Cu^2+^ in an environment with a highly electronegative partner, like oxygen. The signals at ~931 and 950.9 eV are typical for Cu^2+^ and the features at 933.8 and 953.6 eV can be assigned to Cu^+^, respectively^11^,^22^,^23^,^24^,^25^,^26^,^27^,^28^,^29^. The XRD pattern of the spherical particles after synthesis shown in Fig. 2g does not show any evidence on the coexistence of a copper-(I)- and copper-(II)-oxide lattice structure within this sample. Without heat treatment, five obvious diffraction peaks can be found in the pattern, which are indexed to the {110}, {111}, {200}, {220} and {311} planes of cubic Cu_2_O (Fig. 2g,h blue lines, JCPDS card no. 65–3288). This means that the detected Cu^2+^ exists only in amorphous CuO phases or at defect sites within the spherical particles. On the contrary, as shown in Fig. 2e, the as-synthesized octahedral particles show features in the NEXAFS spectrum which can be fully assigned to the chemical nature of copper-(I)-oxide. These differences between the different particle morphologies can only be detected by NEXAFS spectroscopy. Beam damage as a reason for this can be excluded because possible beam induced reactions play a minor role in the investigation of hard-condensed matter like inorganic colloidal particles. Additionally, the vacuum environment during investigation prevents the sample from oxidation and the soft x-rays (<1 keV) do not have the energy needed for crystal rearrangement. Therefore, the found differences between both morphologies can be related to the nature of the immediate neighbors and the chemical state of the investigated element. Furthermore, this may be a factor contributing to the different behavior of the spherical particles in comparison to the octahedrons, which is further investigated in the ESI.

After oxidation both types of particles reveal a complete solid-state transformation from Cu_2_O to CuO, which is demonstrated by the NEXAFS spectra shown in Fig. 2f and with the XRD patterns in Fig. 2h. During the oxidation, the oxygen containing PEG in the samples is removed, which can be confirmed by the TXM images in Fig. 2c,d. Additionally, the spectrum of the O-*K*-edge in Fig. 2f shows no evidence of any other oxygen containing substance. All the recorded features in the O-*K*- and Cu-*L*_*2,3*_-spectra for the spherical as well as the octahedral particles can be assigned to copper-(II)-oxide. The XRD patterns of the spherical (purple) and octahedral (dark cyan) nanoparticles after annealing are shown in Fig. 2h. After the annealing step, the original Cu_2_O signals disappear completely, while new peaks appear, which correspond to the {110}, {002}, and {111} planes of CuO (Fig. 2g and h beige-colored lines, JCPDS card no. 45–0937). This is further evidence that the Cu_2_O nanoparticles have completely transformed to CuO nanoparticles via heating. Analyzing the peak widths using the Scherrer equation yields a crystallite size of 21.8 ± 0.8 nm for the octahedral particles (for 0.3 M NaOH), and 9.5 ± 1.6 nm for the spherical Cu_2_O particles (0.02 M NaOH), whereas the respective particle sizes are ~1000 nm and ~305 nm (analyzed by TXM consistent with the results from dynamic light scattering investigation).

### Spatially resolved surface reactions

**Figure 3:**
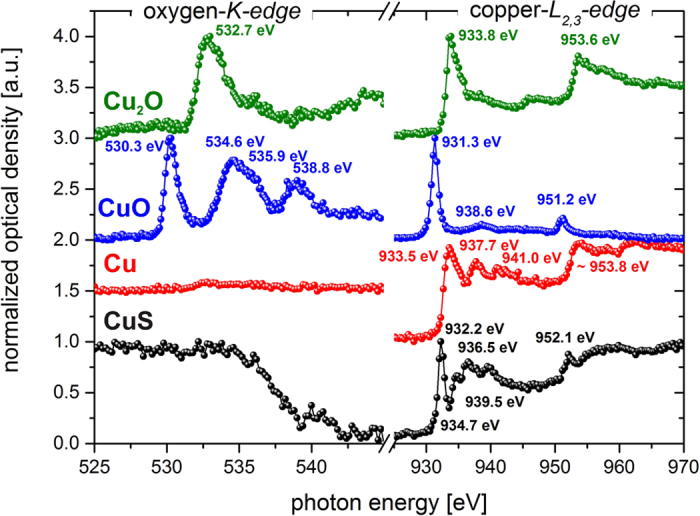
NEXAFS-spectra at the *O-K*- and *Cu-L2,3* edge of reference materials. Black: copper-(II)-sulfide; red: copper; blue: copper-(II)-oxide; green: copper-(I)-oxide. The signal at 932.2 eV in the CuS sample is attributed to the transition of Cu 2p3/2 to 3d states and shows a distinctly different behavior when compared to CuO. The *O-K* edge demonstrates the fundamental differences even more clearly since it does not show a clear absorption edge, which means that there is no oxygen present in the bulk of the reference material.

The currently used NEXAFS-TXM setup at the electron storage ring BESSY II does not allow *in-situ* measurements because of the vacuum setup. Therefore, we have opted for an exemplary gas-surface reaction that is non-reversible at room temperature in normal atmosphere to demonstrate the potential of our approach. Namely, we have used the known, highly specific reaction of CuO towards H_2_S exposure. Even in oxygen depleted atmospheres^30^,^31^ the exothermic reaction^32^:


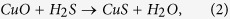


causes fundamental changes in the electrical behavior of gas sensitive layers. At temperatures below 200 °C this conversion is irreversible which means that the reaction product is frozen and samples may be transferred to the NEXAFS-TXM setup without altering the composition of the surface or bulk. Especially since the CuO crystal is transformed to CuS the presence of surface adsorbed species on either CuO or CuS surfaces do not limit the validity of the NEXAFS-TXM results in the low temperature regime. In this operational mode the CuO layer may be used to determine the H_2_S concentration by using a H_2_S induced percolation phase transition and measuring the time t_Percol_ necessary to establish a conducting path via emerging CuS clusters. A second, competing reaction governs the CuO layer’s behavior for high temperatures^33^,^34^,


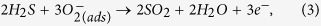


i.e. the layer shows a typical reaction towards reducing gases. In order to confirm the proposed reactions at the employed gas-sensitive materials using the Cu-*L*_*2,3*_ edge and the O-*K* edge we have taken the respective spectra of the reference materials Cu, CuS, CuO, and Cu_2_O. This way we can demonstrate the formation of CuS even without accessing the S-*K* edge, which is unavailable at the currently used beam line. The reference spectra are depicted in Fig. 3 and a detailed discussion thereof can be found in the ESI.

**Figure 4:**
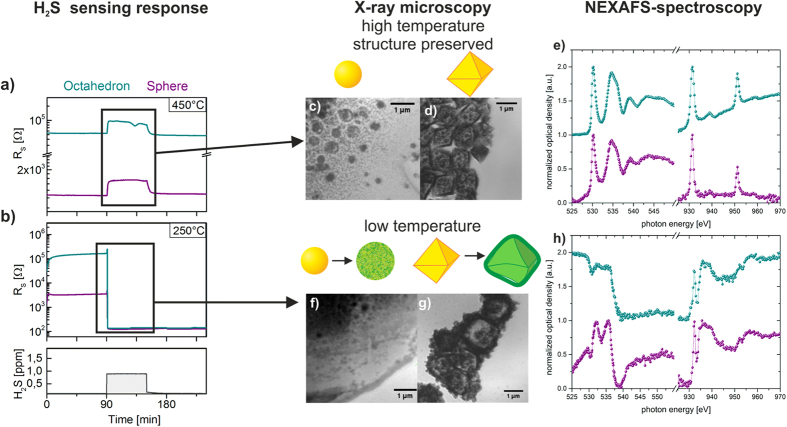
Sensing responses towards hydrogen sulfide in two temperature regimes. **(a)** At 450 °C the sensors show a chemisorption induced increase in electrical resistance. (**b**) At 250 °C stable CuS structures form resulting in a sharp resistance decline without recovery of the sensing signal. Using NEXAFS in combination with x-ray microscopy all surface processes during gas sensing have been investigated spatially resolved. For high temperature operation upon H_2_S exposure the CuO short-range order remains unaltered (**c**) and (**d**) and the NEXAFS spectra for both kind of particles does not change **(e)**, while in the low temperature regime the structure of the nanoparticles changes dramatically **(f,g)**. (**h**) In this temperature regime the CuS structures appear which is confirmed via spectroscopy of the Cu-*L*_2,3_ and O-*K* edge, respectively.

In our experiments both CuO shapes have been exposed to low levels of hydrogen sulfide in the same temperature interval. To evaluate the gas sensitive behavior of the layers the electrical conductivity is determined using interdigitated electrode structures, which form part of a micro-machined hotplate device^35^. The combination of inkjet printing technology and the hotplate device allows for quickly testing novel nano-sized gas sensitive materials. Using NEXAFS-TXM compatible mounts, the experimental conditions have been duplicated in order to monitor all steps of the gas-surface reaction. Figure 4 shows both, the gas sensitive response of the CuO layer and the corresponding NEXAFS-TXM analysis, relating the electrical response of the CuO nanoparticles to the chemical processes at the surface and the bulk phase. Fig. 4a,b shows the electrical response of the gas sensitive layer for both shapes and at temperatures of 450 °C and 250 °C for 1 ppm of H_2_S, respectively. At the high temperature of 450 °C, both particle morphologies react in accordance with equation (3), i.e. the resistivity of the layer increases upon exposure to H_2_S. In the low temperature regime both shapes exhibit a steep decline in resistivity because H_2_S exposure causes the emergence of a continuous path of highly conducting CuS. Since the reaction towards H_2_S is unique and stable CuS structures are formed for temperatures at or below 250 °C the system used in this work offers an ideal test case to demonstrate the feasibility of directly monitoring chemical reactions of gas sensitive materials. The corresponding NEXAFS-TXM analysis is depicted in Fig. 4e,h. In the high temperature regime the morphology of the nanoparticles remains stable which is verified by NEXAFS-TXM analysis of the layer after exposure to H_2_S at 450 °C shown in Fig. 4e. The NEXAFS spectra in Fig. 4d reveal that the chemical composition of the bulk phase is not changed due to the H_2_S atmosphere in this temperature regime. All detected signals of the NEXAFS spectra at the O-*K*- and Cu-*L*_*2,3*_-edge can be assigned to the signals of copper-(II)-oxide and are identical to the spectra prior to gas exposure (compare Figs 2 and 3). This means that only the surface of the nanoparticles reacts during the gas sensing in the high temperature regime.

In stark contrast to this, the morphology and the chemical composition of the particles change if they are exposed to H_2_S in the low temperature regime which is demonstrated by the NEXAFS-TXM in Fig. 4f,g. The detected signals at the O-*K*-edge of both kinds of particles are shown in Fig. 4h, which can be no longer assigned to CuO bonds. This means that the oxygen lattice of the particles is disturbed and differences have to be assigned to the different reaction kinetics of spherical and octahedral particle morphology. The reaction described in equation (2) is not limited to the surface but rather takes place in the bulk phase as well. Thus, the CuO is completely converted into copper sulfide in the low temperature regime. This is underlined by the detected signals at the Cu-*L*_*2,3*_-edge at 932.3 eV and 952.1 eV which can be assigned to CuS (compare Ref. 29 and Fig. 3). However, the detected signals in the region between 934 and 941 eV of the Cu-*L*_*3*_-edge are not in complete accordance with the copper-(II)-sulfide structure. It cannot be ruled out that a distinct amount of Cu_2_S is also formed under these experimental conditions. By comparing the spectra in Fig. 4h to the reference spectra of CuS in Fig. 3 and to the literature^29^ this is further emphasized. Therefore, measurements at the S-*K*-edge would have to be carried out which is not possible at the used TXM at the moment. Additionally, measurements at other x-ray microscopes at the sulfur-*K*-edge with similar spatial resolution allowing single particle analysis are currently not available.

**Figure 5:**
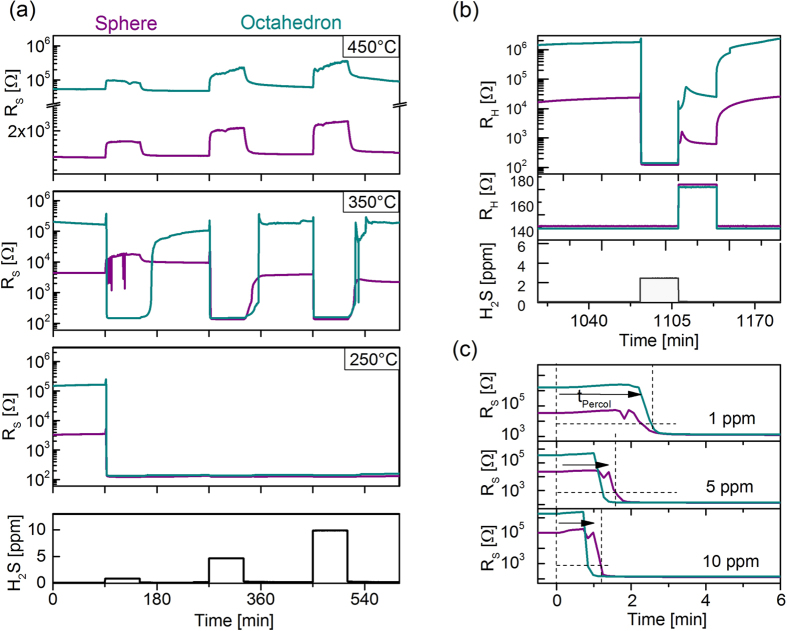
**(a)** Sensing response towards hydrogen sulfide for different temperatures and concentrations. At 450 °C the sensors show a chemisorption induced increase in electrical resistance as a function of the H_2_S concentration. At 350 °C the behavior differs for spherical and octahedral particles at 1 ppm. For higher concentrations the formation of CuS structures becomes dominant, yet unstable, as the sensing resistance recovers after exposure. At 250 °C the CuS structures are stable on the surface and no recovery of the sensing signal is observed. **(b)** However, applying a heating protocol the sensor characteristics can be reproducibly recovered. The temperature is indicated in the middle panel by depicting the corresponding heater resistivity R_H_. **(c)** Measuring t_Percol_ enables selectively determining the H_2_S concentration using the trace gas induced phase transition from semiconducting to conducting overall behavior.

Because temperature apparently plays a pivotal role in the behavior of the CuO layers we have expanded the gas sensitive measurements to intermediate temperatures and further concentrations. The experimental results are depicted in Fig. 5. Lowering the temperature to 350 °C both reaction strengths are on the same order of magnitude which is highlighted by the shape dependent response to 1 ppm H_2_S. In order to further investigate the shape and size dependent gas reaction of CuO further experiments have been performed. The results are presented in the ESI. While sphere-based layers still react according to equation (2), i.e. by increasing the electrical resistivity upon gas exposure, octahedron-based layers undergo the percolation phase transition. Increasing the applied H_2_S concentration above 1 ppm causes the process described by equation (2) to become dominant for both shapes, i.e. the percolation phase transition always takes place. Nevertheless, when removing H_2_S at 350 °C the CuS structures recede and both the spherical as well as the octahedral layer return to p-type semiconductivity. Lowering the temperature to 250 °C, the conversion to conductive behavior becomes permanent, i.e. even after removing H_2_S from the test chamber the CuS structures remain stable as indicated by the stable, low resistivity which can be seen in Fig. 4b. Consequently, a temperature treatment might be used to reset the functional layers. This approach is demonstrated in Fig. 5b where the temperature of the gas sensitive layer is increased to 450 °C after the end of the gas exposure. This is indicated by the temperature dependent resistivity of the microheater R_H_ in the center panel of the graph. By applying 450 °C to the layer for 30 min it returns to p-type semiconductivity thus resetting the original gas sensing conditions. Fig. 5c shows the behavior of the layers after the onset of the exposure to a concentration of 1 ppm, 5 ppm, and 10 ppm H_2_S, respectively, at 250 °C in greater detail. It demonstrates how the percolation time t_Percol_ may be used to infer the H_2_S concentration. To characterize the layer’s behavior the time t_Percol_ is defined as the time from the onset of gas exposure until the resistivity of the layer drops below the threshold value of 300 Ω. This novel approach may be used to determine the H_2_S concentration in a fundamentally new, highly specific way because only H_2_S causes the percolation phase transition.

### Imaging local surface reactions

**Figure 6:**
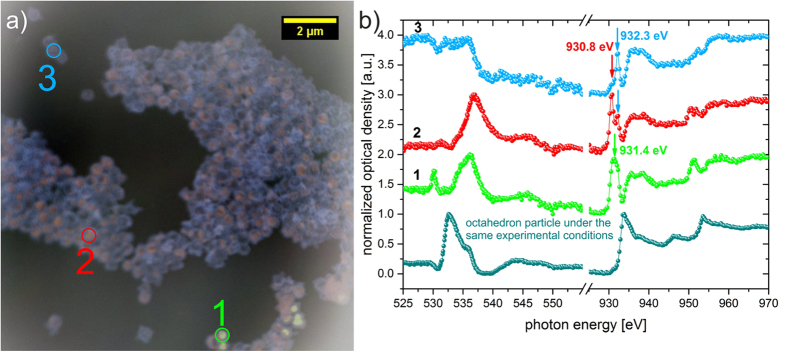
**(a)** TXM-micrographs of the spherical particles after printing and direct exposure to H_2_S at 200 °C (overlay 930.8 eV (red), 931.4 eV (green), 932.3 eV (blue)). The false color representation is used as a tool here in order to visualize the chemical inhomogeneity of the sample. Each color corresponds to a particular type of chemical bond. This information is extracted from the spectrum of each pixel of the image. **(b)** NEXAFS spectra of the O-*K*-edge and the Cu-*L*_*2,3*_-edge of the marked particles in **(a)** are represented in green, red and blue and are labeled 1, 2, and 3, respectively. For comparison, the spectrum of an exemplary octahedron particle under similar experimental conditions is depicted as well. The arrows in **(b)** indicate the location of peaks and photon energies used to characterize the different chemical species in **(a)**.

Because the gas surface reactions crucially depend on the metal oxide material we also tested the as-synthesized Cu_2_O nanoparticles with respect to their gas sensitivity. Unfortunately, due to remaining PEG the sensing layer resistances composed of Cu_2_O are in excess of 10^8^ Ω making the particles unsuitable for use as gas sensing material. In order to assess the influence of H_2_S exposure on Cu_2_O particles a NEXAFS sample was produced without using the inkjet printing system, i.e. without using PEG. Future work will entail tuning the rheological properties without using PEG thus allowing for an electrical read-out of inkjet printed Cu_2_O layers. To investigate the gas-surface reactions now, the as-synthesized particles are exposed to 5 ppm H_2_S in dry synthetic air for 2 hours at 200 °C. Afterwards, the chemical composition of the particles is examined by NEXAFS-TXM. Fig. 6a shows a resulting TXM-micrograph of the spherical particles, which is given in false color representation to directly map the chemical inhomogeneity of this sample after the H_2_S exposure experiment. The spatially resolved spectroscopy approach allows for extracting information about the chemical bonds and oxidation state prevailing in each pixel of the image. The false colors are assigned subject to the marked peak position in Fig. 6b at the Cu-*L*_*2,3*_-edge. The red color in Fig. 6a is assigned to photon energy 930.8 eV, which means that at this energy a chemical bond of Cu^2+^ in an oxygen environment will show a maximum of contrast in x-ray absorption whereas the other copper components show a much smaller absorption. The blue color in Fig. 6a is assigned to photon energy 932.3 eV, which is the characteristic absorption energy of Cu^2+^ in a sulfide environment. The green color presents a transition state between the oxygen and sulfide environment of Cu^2+^. This cannot be detected in a conventional x-ray absorption spectroscopy experiment with a spot size of a few microns. The NEXAFS-TXM method employed here features a spatial resolution on the order of 20 nm and demonstrates how NEXAFS-TXM helps uncovering the underlying surface processes. The respective NEXAFS spectra of the marked particles in Fig. 6a are shown in Fig. 6b. Additionally, a representative NEXAFS spectrum of an octahedron particle prepared under similar experimental conditions as the spherical particles is shown in Fig. 6b. The corresponding TXM-micrograph is presented in ESI Figure S1.

The analysis of the NEXAFS spectra has utilized the O-*K*-edge in the energy range 525 eV–555 eV as well as at the Cu-*L*_*2,3*_-edge in the energy range 925 eV–970 eV. In this way information about the chemical state of both atomic species constituting the gas sensitive material can be obtained.

The comparison of the spectra of the octahedral particles with literature data and the measured references are in good accordance for copper-(I)-oxide^22^,^23^,^24^,^27^. In detail: The recorded NEXAFS spectra at the O-*K*-edge show the typical pronounced absorption peak at 532.7 eV and minor features at higher photon energy for copper-(I)-oxide. The main absorption feature can be explained by the transition of an electron from the 1 s core level to the final states of *p* symmetry at the oxygen site. The minor features at higher photon energy are caused by the hybridization of the O 2p states with the Cu d and s states^23^. The recorded main signals of the octahedron particles at the Cu-*L*_*3*_-edge (2p_3/2_; around 933.8 eV) and the Cu-*L*_*2*_-edge (2p_1/2_; around 953.6 eV) are ascribed to the electron transition from the Cu p states to the unoccupied 3d level due to the unusual crystal structure of Cu_2_O^22^.

In contrast, the NEXAFS spectra at the O-*K*-edge of all marked spherical particles in Fig. 6a show a significant difference in comparison to the spectrum of the octahedral particles and to the Cu_2_O reference. For the marked particle 3, no distinct absorption signal is recorded, which leads to the assumption that the oxygen lattice structure of the blue area in Fig. 6a is more or less disturbed. The Cu-*L*_*2,3*_-edge spectrum of particle 3 clearly shows that a copper sulfide structure is formed. The peak at 932.3 eV indicates the existence for Cu^2+^ in a sulfide environment, whereas the region between 934 to 940 eV does not precisely show the minima and maxima of the pure CuS structure (compare Fig. 3 and the Ref. 29). This means that there is a coexistence of CuS and Cu_2_S. Additionally, the shoulder around 930.8 eV marks the existence of Cu^2+^ in an environment with an electronegative anion, like oxygen^27^. Similar spectra can be obtained for all the blue areas of Fig. 6a for both absorption edges. The recorded spectra of particle 1 and 2 presents in our view transitions states in this solid-state transformation process, because the recorded signals for both particles cannot be fully assigned to the Cu_2_O, CuO or to the CuS structure.

These results clearly indicate that the as-synthesized spherical particles undergo a solid-state transformation into copper sulfide upon H_2_S exposure while octahedral particles do show a fundamentally different behavior. This is a direct observation of the profound influence of particle shape on gas-surface reactions. Possibly, the exposed crystallographic plane and the associated surface energies play a central role in this process^36^ and consequently influence on the gas sensitive behavior is expected. Additionally, this example illustrates the high potential for combining the spatial resolution of x-ray microscopy with the chemical information obtained from NEXAFS spectroscopy for functional materials development.

## Conclusion

Because of the highly complex nature of gas-solid interactions and the current lack of possibilities to directly monitor surface reactions the behavior of solid-state gas sensors based on functional metal oxides is inadequately understood. Within this work the use of NEXAFS-TXM as a means to shed light onto chemisorption processes at metal oxide surface has been demonstrated using nanoparticles synthesized using a wet chemistry approach. Here, the combination of a bottom-up approach to produce nanoparticles with well-defined shape and form with the inkjet technology proved to be a promising method to directly apply basic research results in sensorial devices. The NEXAFS-TXM samples have been prepared copying all steps of the gas detection, i.e. after synthesis of Cu_2_O, after transformation to CuO, after exposure to H_2_S at low temperatures and after resetting the functional layer, i.e. after converting CuS back to CuO. Using narrow bandwidth x-ray absorption spectroscopy it was possible to reveal the underlying changes to the material system, morphology and chemical composition. The reaction between CuO/Cu_2_O surfaces and H_2_S has provided a suitable sample system since the surface states of the different reactions may be retained for examination in the HZB NEXAFS-TXM. Consequently, the existing explanations for changes in electrical conductivity observed during the gas characterization could be substantiated using spectral analysis of the Cu-*L*_*2,3*_ and O-*K* edges. In particular, the NEXAFS-TXM analysis demonstrated that for temperatures below 250 °C CuO is converted to CuS whenever H_2_S is present. The emergence of highly conducting CuS structures as well as the chemical structure of CuO have been imaged spatially resolved revealing the sensing mechanism responsible for the selective detection of H_2_S. This process is not limited to the particle surface but encompasses the whole particle thus completely destroying the CuO crystalline structure. Furthermore, the transformation is reversible in the absence of H_2_S and applying temperatures of 450 °C to the layer. This makes the reaction mechanism a suitable starting point for a new type of gas sensor based on the percolation effect induced by H_2_S. Future setups based on this approach should offer the possibility for *in-situ* measurements under real-world conditions by combining a gas measurement apparatus with a NEXAFS setup, thus enabling a spatially resolved imaging of the chemisorption processes at metal oxide surfaces under gas exposure.

## Methods

### Dynamic light scattering

Particle sizes were determined by dynamic light scattering (DLS), using a He:Ne gas laser (λ = 632.8 nm, 22.5 mW) and a goniometer (ALV/SP-86; from ALV Company, Langen, Germany) equipped with an index match bath. As detector two Perkin Elmer photomultipliers (SPCM-CD2696 Rev.G) were used and cross correlated by an ALV 5000/E correlator. The intensity auto-correlation function was transformed into the intermediate scattering function by using the Siegert relation. Diffusion coefficients were calculated by cumulant analysis and used to determine the hydrodynamic radii via the Stokes–Einstein equation. The measurements were performed at an angle of 100°.

### Synthesis of Cu_2_O nanoparticles

Cu_2_O nanospheres with radii ranging from 150 nm to 1600 nm were prepared with high yield needed for inkjet printing. In the synthesis protocol Cu(NO_3_) 2•3H_2_O was dissolved in a 1000 ml three-necked flask in 1/3 of the total water (500 ml) and then kept stirring for about 30 min at 250 rpm. Meanwhile 5 g Polyvinylpyrrolidone (PVP, average MW=55000) were dissolved in the remaining water. After complete dissolution the PVP solution was added dropwise to the Cu(NO_3_) solution and the reaction mixture was then stirred for another 2 hours. Then 453 μl of hydrazine solution (35% N_2_H_4_ in water) was added to the reaction mixture drop-wisely. The color of the solution changed into orange immediately after the introduction of N_2_H_4_, which indicates the production of Cu_2_O nanoparticles. The resulting solution was kept stirring for 30 min at room temperature and was then washed with H_2_O and EtOH several times by centrifugation and redispersion (Multifuge 3 SR, Heraeus: 900 g, 45 min, 21 °C). In a final step the Cu_2_O nanoparticles were redispersed in a small amount of EtOH and stowed away in the dark until further use.

To further optimize the synthesis for higher yield this standard protocol was modified by the addition of NaOH. To achieve the wanted NaOH concentration in the reaction mixture water in the Cu(NO_3_) solution was reduced by a certain amount to be later on replaced by the same volume of a 2 mol/l NaOH stock solution. After drop-wise addition of the PVP solution the reaction mixture was stirred for 2 hours and then the NaOH stock solution volume added under stirring (250 rpm). The N_2_H_4_ was then added after 5 min further stirring. The remaining protocol was the same as in the standard synthesis without NaOH.

### Inkjet printing

After washing the Cu_2_O particles are dispersed in pure ethanol and 60 s of ultrasound are applied prior to printing in order to obtain a homogeneous dispersion. 2 ml of the dispersion are transferred into the printer cartridges. The dispersions containing spherical particles were modified with 0.5 ml PEG 400 to improve printability.

The inkjet deposition is performed using a DIMATIX DMP 2831 printer system with 10 pl print heads. The nozzle is kept at room temperature and the substrate at 30 °C. The nozzle droplet firing voltage was set to 27 ± 3 V. The drop-to-drop distance (pitch) was set to 40 μm. For gas sensor devices 6 stacked layers were deposited. For NEXAFS measurement particles were deposited on a silicon nitride membrane. Sample preparation printer parameters have been identical for gas sensor devices and NEXAFS-TXM samples.

### Conversion of Cu_2_O to CuO

For the gas sensing experiments Cu_2_O particles were converted on-chip by the integrated heaters at a temperature of 350 °C for several hours prior to exposing the sensors to trace gases. This is well above the limit for stable Cu_2_O.

For the NEXAFS-TXM characterization the Cu_2_O particles were oxidized in dry ambient air in a quartz tube furnace (ATV PEO 603) by linearly ramping the temperature for 1 h to the destination temperature of 400 °C and holding it for 1 h before cooling down.

### XRD

X-ray diffraction measurements were operated in the 2θ range from 20 to 80° on a Bruker D8-Advance X-ray diffractometer with Cu Kα1 radiation (λ=1.5406 Å). The radiation source was operated at a voltage of 40 kV and a current of 40 mA. The step size of the measurements is 0.02° with an integration time of 3.5 seconds per step.

### Sample preparation of reference-samples for NEXAFS-TXM

The copper grids with carbon support film (200 meshes, Science Services, Munich, Germany) have been pretreated by 10 s of glow discharge. Reference materials for Cu_2_O (Sigma-Aldrich; product no. 566284; purity >99.99%), CuO (Sigma-Aldrich, product no.203130; purity >99.999%) and CuS (Sigma Aldrich, product no. 450820, purity >99.99%) are commercially ordered. These high purity materials are delivered in a glass ampule, which is completely sealed under inert gas atmosphere. The glass ampule was opened directly before the measurement and some crystals of the reference materials have been spread over the prepared grids and directly transferred into the oxidation protective vacuum of the TXM measurement.

A chemical vapor deposition (CVD) process on a TEM grid with holey carbon support film was used to prepare the copper metal reference (film thickness ~30 nm). The sample was stored and placed on the sample holder under argon atmosphere. Only during the time for the transfer from one apparatus to another the sample to the TXM microscope was not protected by inert gas.

### Near edge X-ray absorption fine structure-transmission X-ray microscopy (NEXAFS-TXM)

The NEXAFS-TXM spectra were recorded on the O-*K*-edge and the Cu-*L*_*2,3*_-edge with the HZB-TXM which is installed at the undulator beamline U41-FSGM at the electron storage ring BESSY II, Berlin, Germany. It provides a high spatial resolution close to 10 nm (half-pitch) and a spectral resolution up to E/ΔE ≈ 10^4^. Typical spectra are presented for each set of measurements. The TXM allows measurements to be taken at room or liquid nitrogen temperature in a vacuum of 1.3 × 10^−9^ bar. The spectra were recorded at room temperature in transmission mode by taking a sequence of images over a range of photon energies covering the investigated absorption edges with a calculated E/ΔE > 5800 for the Cu-*L*_*2,3*_-edge and E/ΔE > 12000 for the O-*K*-edge. Note that the exit slit of the monochromator was set to 9 μm for the Cu-*L*_*2,3*_-edge and 7 μm for the O-*K*-edge resulting in the given calculated monochromaticity values. The exposure time for one image with 1340 × 1300 pixels was 24 s for the Cu-*L*_*2,3*_-edge and 4 s for the O-*K*-edge to achieve a sufficient signal to noise ratio in the images. Taking an image stack with up to 226 images at different energies needs inherently about 45 to 120 min because of all necessary movements, exposure time, and camera read out time and image storage. The NEXAFS spectra were normalized since the photon flux varies as a function of photon energy (hν) and time in the object field (x, y). The normalization was performed by dividing the intensity I(x, y, hν) recorded on a single nanostructure by the intensity I_0_ (x + Δx, y + Δy, hν) recorded in its sample free proximity at position (x + Δx, y + Δy). Both I(x, y, hν) and I_0_(x, y, hν) were recorded within the same image stack since bare regions in the vicinity of the nanostructures permit the measurement of I_0_.

The image stacks were aligned and analyzed with the aXis2000 software^37^.

### Gas sensitive characterization

The apparatus used to control the temperature of the functional MOX layers as well as the composition of the gas matrix is described in detail in^38^. Here, the read-out frequency was set to 1 Hz, the temperature of the microelectromechanical platform was controlled via the heaters resistivity value after the relation between temperature and resistance has been established^35^.

## Additional Information

**How to cite this article**: Henzler, K. *et al.* Investigation of reactions between trace gases and functional CuO nanospheres and octahedrons using NEXAFS - TXM imaging. *Sci. Rep.* **5**, 17729; doi: 10.1038/srep17729 (2015).

## Supplementary Material

Supplementary Information
