# Supplementary material for: Investigation of reactions between trace gases and functional CuO nanospheres and octahedrons using NEXAFS-TXM imaging
Source: Sci Rep. 2015 Dec 3;5:17729. doi: 10.1038/srep17729 (PMC4668554; doi:10.1038/srep17729)
Supplement: Supplementary Information [file srep17729-s1.pdf]

Electronic Supplementary Information

Investigation of reactions between trace gases and functional CuO nanospheres and octahedrons using NEXAFS - TXM imaging

Katja Henzler[1,2], Axel Heilmann[3,4,5], Janosch Kneer[5], Peter Guttmann[1], He Jia[1], Eckhard Bartsch[3,4], Yan Lu[1], Stefan Palzer[5]

[1] Institute for Soft Matter and Functional Materials, Helmholtz-Zentrum Berlin für Materialien und Energie GmbH, Hahn-Meitner-Platz 1, 14109 Berlin, Germany

[2] Present address: Laboratory for Synchrotron Radiation – Catalysis and Sustainable Chemistry, Paul Scherrer Institut, 5232 Villigen PSI, Switzerland

[3] Institut fur Makromolekulare Chemie, University of Freiburg, 79104 Freiburg, Germany

[4] Institut fur Physikalische Chemie, University of Freiburg, 79104 Freiburg, Germany

[5] Laboratory for Gas Sensors, Department of Microsystems Engineering, University of Freiburg, Georges-Köhler Allee 102, 79110 Freiburg

Correspondence and requests for materials should be addressed to S.P. (email: Stefan.palzer@imtek.uni-freiburg.de)

TXM-image of octahedral particles after H_2S exposure

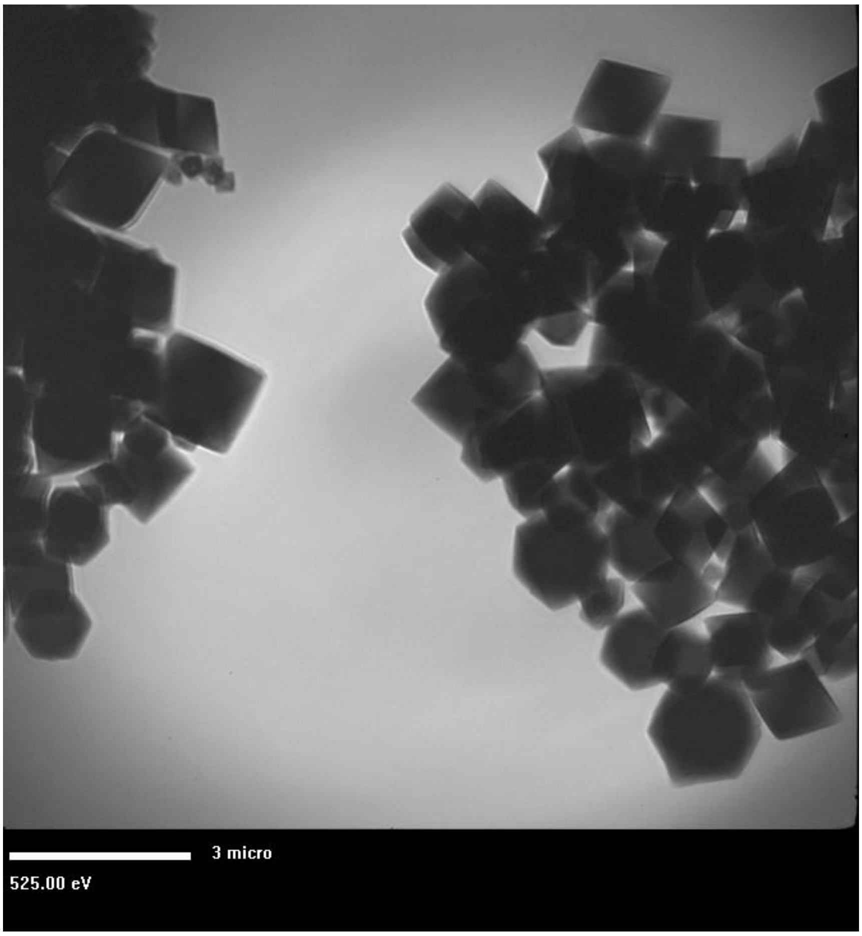

Figure S1 TXM-micrograph of the octahedral particles after direct exposure to H_2S at a photon energy of 525 eV.

Scanning electron microscopy image of CuO before and after 12 H_2S measurement cycles using the percolation phase transition

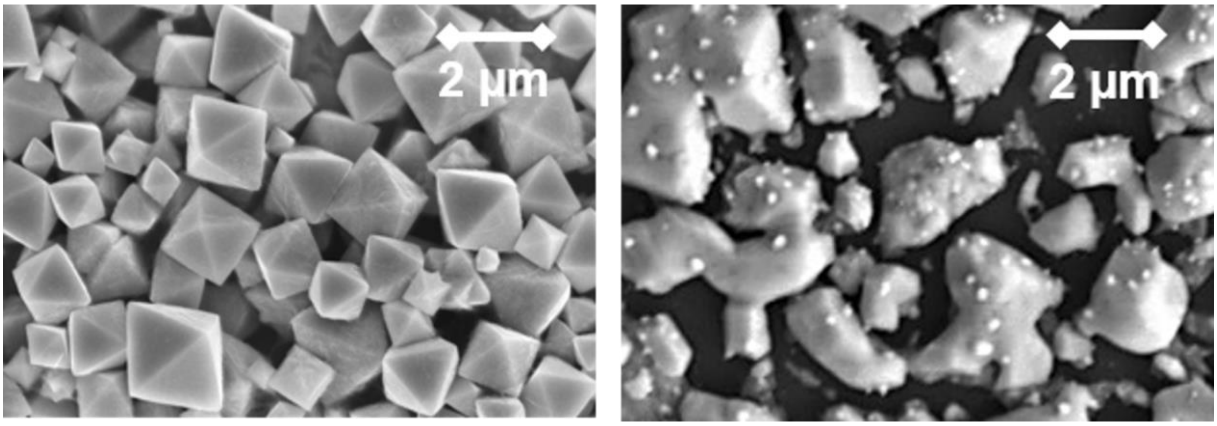

Figure S2 Scanning electron microscopy image of particles as deposited (left) and after (right) 110 h of operation including 12 cycles of conversion from CuO to CuS and back, i.e. total of 160h exposed to H_2S.

Discussion of the reference material spectra

Figure 3 of the manuscript shows the NEXAFS spectra of the measured reference materials. In the following paragraph the measured spectra are compared to the available literature data of this compounds. In a brief comparison they are in good accordance. In detail:

Copper-(II)-oxide:

Cu-$L_{2,3}$-edge:

The Cu 2p spectrum shows two doublets separated by about 20 eV due to the spin orbit splitting. The ground state of CuO can be described as a mixture of $3d^9$ and $3d^{10}$ $\underline{L}$ character, where $\underline{L}$ stands for a hole in the O 2p band[1,2,3,4,5,6]. The main peak for copper-(II)-oxide around 931 eV ($2p_{3/2}$) corresponds to a $2p^5 3d^{10}$ final state. In Ref. 6 the main peak of the Cu-L_3-edge is given at 930.8 eV (measured with an energy resolution of 200 meV, grating monochromator, total fluorescence yield), whereas the same signal in Ref. 1 is given for photon energy of 931.3 eV (measured with an energy resolution of 400 meV, beryl double-crystal monochromator, total yield mode). Literature data for CuO measured in transmission mode have not been published up to now. Therefore, small differences between the literature and the actual measurement data can be traced back to these experimental differences. The Cu $2p_{1/2}$ peak appears approximately 20 eV above the Cu $2p_{3/2}$ signal, yet with much lower intensity. This signal is broader due to the shorter core-hole lifetime and the interaction with the $2p_{3/2}$ continuum.

O-K-edge:

The spectrum can be divided into two regions. The first signal at 530.3 eV can be attributed to the transition of the oxygen 1s to the $3e_g$ orbital[7] . The $3e_g$ orbital originates from the hybridization of the O 2p and Cu 3d states. The second region 4-10 eV above the threshold is attributed to oxygen p character hybridized with metal 4s and 4p states[7].

Copper-(I)-oxide:

Cu-$L_{2,3}$-edge:

The spectrum of Cu_2O clearly distinguishes from the CuO spectrum. The main lines of the Cu_2O spectrum can be found at higher photon energies (933.8 eV and 953.6 eV). The signal at 933.8 eV is asymmetric and followed by a plateau with weak structure on the high-energy site. This can be explained by a fraction of d character in the unoccupied states. The amount of unoccupied d character is related to the unusual structure of Cu_2O, in which the Cu is linearly coordinated to two oxygen atoms[1].

O-K-edge:

The O-K-edge shows the typical pronounced absorption peak at 532.7 eV and minor features at higher photon energies. The main absorption feature can be explained by the transition from the 1s core level to the final states of p symmetry at the oxygen site. The minor features at higher photon energies are caused by the hybridization of the O 2p states with the Cu d and s states[2].

Copper-(II)-sulfide:

Cu-$L_{2,3}$-edge:
The signal at 932.2 eV is attributed to the transition of Cu $2p_{3/2}$ to 3d states. The equivalence of all Cu sites in CuS is considered in terms of oxidation, that one-third of all Cu sites can be in the Cu(II) state [1,8]. Therefore, the oxidation state of Cu in CuS is generally assigned to Cu(I) with the electronic configuration $3d^{10}$ - a filled 3d shell[8]. However, the simulations in Ref. 8 clearly demonstrate that there must be an increased contribution of 3d states and thereby the presence of Cu(II) sites. The absorption feature between 934 to 940 eV is attributed to an excitation of the 2p electrons into the conductive band[8].
O-*K*-edge:
To the best of our knowledge this spectrum is not to be found among the available literature. It does not show a clear absorption edge, which means that there is no oxygen present in the bulk of the reference material. However, adsorbed oxygen and CO_2 molecules at the material surface cannot be completely excluded due to sample preparation at normal atmosphere.

Copper metal:
Cu-$L_{2,3}$-edge:
The recorded NEXAFS signals for the Cu metal sample show a different absorption behavior due to the full 3d states and thereby the formal valence of 0. The $L_{2,3}$-edge exhibits a step-like behavior, with an around 3 times weaker absorption intensity in absolute scale. This is explained by the smaller matrix elements for the 2p-4s transition and the broad 4s band. Additionally, the 4s wave function is more extended than the 3d orbital; therefore the core-hole effect becomes negligible[1,2].
O-K-edge:
The recorded spectrum of the Cu metal sample shows no evidence that the sample was oxidized by atmospheric oxygen during the sample transfer.

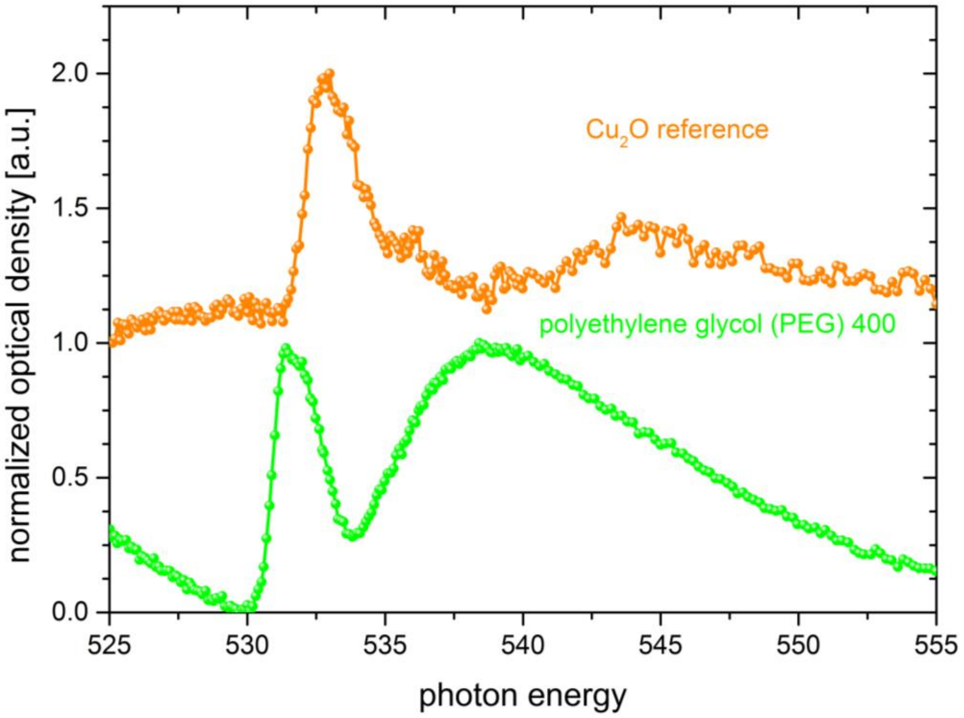

Figure S3 NEXAFS-Spectra of the O-*K*-edge of a copper-(I)-oxide reference (orange) and the used polyethylene glycol (PEG) 400 (green) to tune the rheological properties of the particle ink.

Influence of shape in gas sensitivity

The gas sensitive behavior of functional metal oxides can be tailored by controlling both size and shape of the particles. This influence on the sensitivity has been investigated in the temperature regime between 250°C - 450°C in dry synthetic air using relevant trace gases in a range of concentrations. In particular, the gas-induced changes in the electrical resistivity of the two shapes of CuO particles towards the reducing gases hydrogen (H_2) and ammonia (NH_3) have been tested. To this end, spherical particles obtained by adding 0.03 M NaOH and octahedral particles obtained by adding 0.3 M NaOH (following the synthesis route described in the main text) have been used in the gas exposure experiments. In order to reliably compare the gas reaction of differently shaped particles of comparable size, both types have been characterized simultaneously using a fully automated apparatus capable of controlling and reading-out up to 8 sensorial devices in parallel[9].

Figure S4 shows the results of the gas sensitive characterization for hydrogen (H_2) and ammonia (NH_3). Layers composed of the spherical particles show considerable higher electrical conductivity resulting in a baseline resistivity that is about one order of magnitude below that of the octahedron based layers. To investigate the sensitivity the devices have been exposed to varying levels of hydrogen concentrations ranging from 0 - 400 ppm. The detection of hydrogen occurs mainly via chemisorbed oxygen species on the CuO surface as per the following net reactions[10]:

$$2H_2 + O^{-}_{2(ads)} \rightarrow 2H_2O + e^{-} \quad , \qquad (1a)$$

$$2H_2 + O^{2-}_{2(ads)} \rightarrow 2H_2O + 2e^{-} \quad , \qquad (1b)$$

depending on the prevailing, temperature-dependent chemisorbed oxygen species. Consequently, for p-type semiconducting CuO layers the resistivity increases due the release of electrons into the CuO layer. Interestingly, for spherical particles the sensitivity is almost temperature independent, whereas for octahedral particles the sensitivity is highest for operational temperatures of 350°C. This is an indication that the chemisorbed oxygen species depend on the exposed crystallographic plane as recently suggested [11] and the results presented therein regarding octahedral nanoparticles.

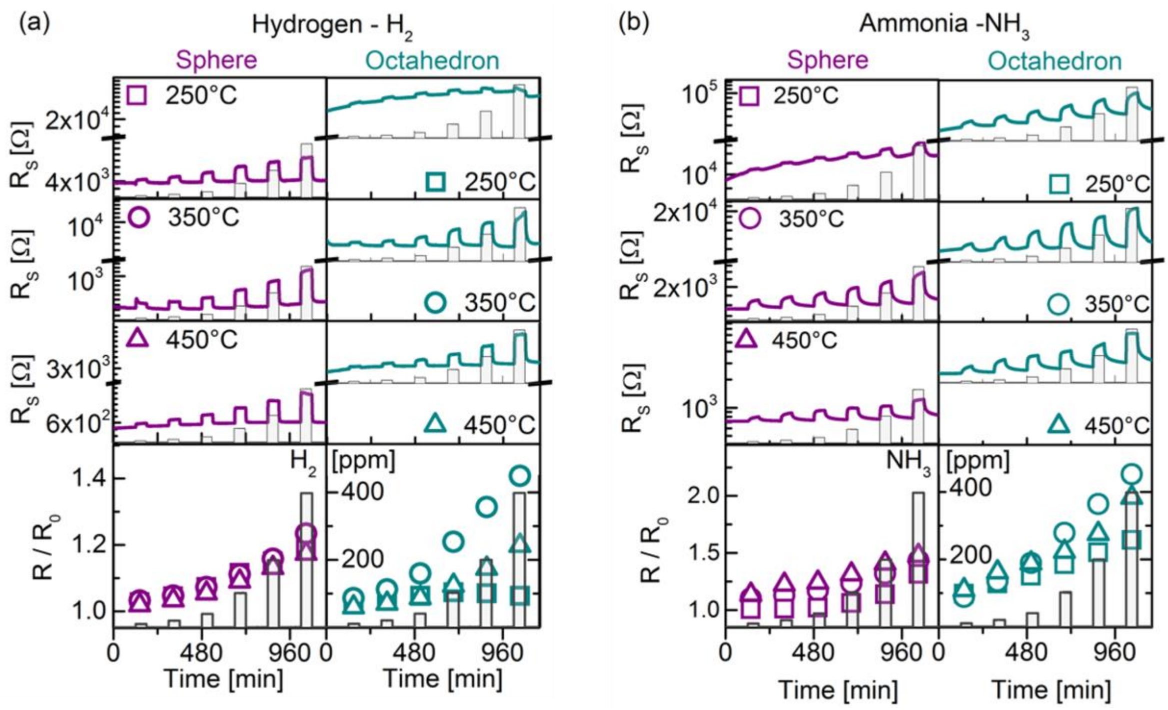

Figure S4 Sensing characteristics towards different concentrations of hydrogen and ammonia. The CuO layers are composed of spheres with a mean diameter of 200 nm and octahedrons of size ~1000 nm, respectively. The sensor reaction R/R_0 has been calculated from the experimental data after performing a baseline fit to correct for long-term drift of the layer's resistance and are indicated by the symbols corresponding to each temperature in the bottom graphs, respectively. The bars in the bottom of both figures represent the applied gas concentration. **(a)** Sensor reaction towards varying concentrations of hydrogen for different temperatures in dry synthetic air. The reaction of the layers based on spherical particles is depicted on the left hand side (violet) while the octahedron-based layers are shown on the right hand side (dark cyan), respectively. **(b)** The corresponding reactions towards varying levels of ammonia.

The layer's response towards different concentrations of ammonia gas in the same temperature interval, depicted in Fig. S4b, yields further evidence of the importance of shape in gas sensing applications for functional metal oxide particles. While the detection mechanism and surface chemistry are more complex the sensitivity is still influenced to a large extend by the chemisorbed oxygen species and the net reaction ultimately influencing the resistivity reads[12]:

$$4NH_3 + 3O^{-}_{2\,(ads)} \rightarrow 2N_2 + 6H_2O + 3e^{-} \quad , \qquad (2a)$$

$$2NH_3 + \frac{3}{2}O^{2-}_{2\,(ads)} \rightarrow N_2 + 3H_2O + 3e^{-} \quad . \qquad (2b)$$

However, the detection mechanism undergoes several intermediate steps with ammonia molecules directly adsorbing on the metal oxide surface. For this reason the reaction towards NH_3 and intermediate adsorbed species is not fully reversible at temperatures below 350°C since ammonia and intermediate molecules remain adsorbed on the surface. Nonetheless, and in accordance with the results for hydrogen, the sphere-based layer's sensitivity shows little temperature dependence whereas the octahedron-based layer shows the highest sensitivity at 350°C. While for equally shaped

particles the sensitivity usually increases for a decreasing size due to a higher surface to volume ratio, here the influence of shape of on the gas sensitive behavior governs the behavior of the layer.

---

[1] Grioni, M. *et al.* Studies of copper valence states with Cu L_3 x-ray-absorption spectroscopy. *Phys. Rev. B* **39**, 1541 (1989).
[2] Grioni, M., van Acker, J.F., Czyžyk, M.T. & Fuggle, J.C. Unoccupied electronic structure and core-hole effects in the x-ray-absorption spectra of Cu_2O. *Phys. Rev. B* **45**, 3309 (1992).
[3] van der Laan, G., Pattrick, R.A.D., Henderson, C.M.B. & Vaughan, D.J. J. Oxidation state variations in copper minerals studied with Cu 2p x-ray absorption spectroscopy. *J. Phys. Chem. Solids* **53**, 1185-1190 (1992).
[4] Koster, A.S. X-ray Lα emission and L_{III} absorption spectra of copper compounds. *Mol. Phys.* **26**, 625-632 (1973).
[5] Ghijsen, J. *et al.* Electronic structure of Cu_2O and CuO. *Phys. Rev. B* **38**, 11322 (1988).
[6] Kvashnina, K.O. *et al.* Electronic structure of complex copper systems probed by resonant inelastic x-ray scattering at Cu_{L3} edge. *Physica B* **404**, 3559-3566 (2009).
[7] de Groot, F.M.F. *et al.* Oxygen 1s x-ray-absorption edges of transition-metal oxides. *Phys. Rev. B* **40**, 5715 (1989).
[8] Vegelius, J.R. *et al.* X-ray spectroscopic study of Cu_2S, CuS, and copper films exposed to Na_2S solutions. *J. Phys. Chem C* **116**, 22293-22300 (2012).
[9] Kneer, J. *et al.* Apparatus to characterize gas sensor response under real-world conditions in the lab. *Rev. Sci. Instrum.* **85**, 055006 (2014).
[10] Morrison, S.R. Selectivity in semiconductor gas sensors. *Sensor. Actuat. B–Chem*. **12,** 425-440 (1987).
[11] Han, X. *et al.* Synthesis of tin dioxide octahedral nanoparticles with exposed high-energy {221} facets and enhanced gas-sensing properties. *Angew. Chem. Int. Ed.* **48**, 9180–9183 (2009).
[12] Shao, F. *et al.* Interaction mechanisms of ammonia and tin oxide: a combined analysis using single nanowire devices and DFT calculations. *J. Phys. Chem. C* **117**, 3520–3526 (2013).
